# ActionX: pre-training action experts with reinforcement learning for vision-language action models

**DOI:** 10.3389/fnbot.2026.1806605

**Published:** 2026-04-14

**Authors:** Tinghao Yi, Quantao Yang, Enhong Chen

**Affiliations:** 1 School of Computer Science and Technology, University of Science and Technology of China, Hefei, China; 2 Department of Robotics, Perception and Learning, KTH Royal Institute of Technology, Stockholm, Sweden

**Keywords:** flow matching, manipulation, reinforcement learning, robotics, VLA

## Abstract

Vision-Language Action (VLA) models have enabled language-driven robotic manipulation by integrating language instructions, visual perception, and action generation. However, existing VLA approaches heavily rely on large-scale human demonstration datasets, which leads to substantial data collection and training costs. To address this, we propose ActionX, a pretraining framework that learns Action eXperts using reinforcement learning while leveraging a frozen, pretrained Vision-Language Model (VLM) backbone. The pretrained action expert is then integrated with the vision-language backbone and fine-tuned end-to-end using a small amount of expert data to align perception, language, and action for downstream manipulation tasks. We evaluate ActionX on the LIBERO and Meta-World benchmarks as well as real-world robotic manipulation scenarios. Experimental results show that ActionX achieves +16% success rate compare to state-of-the-art VLA models trained with large-scale demonstrations, while requiring only less than 100 expert demonstrations for real robot tasks for whole training phase. This performance is achieved by establishing an optimized action expert model through reinforcement learning, which significantly enhances VLA training efficiency.

## 1 Introduction

Vision-Language Action (VLA) models have emerged as a powerful paradigm for language-driven robotic manipulation, enabling robots to translate natural language instructions into actionable sequences by jointly reasoning over visual observations, language commands, and robotic states. This capability allows robots to interact effectively with complex environments and perform tasks that require precise coordination between perception and action. Despite their promise, current VLA approaches (Black et al., 2024; Bjorck et al., 2025; Zhang X. et al., 2025) rely heavily on large-scale human demonstration datasets for pre-training. Collecting such datasets is costly, time-consuming, and often impractical for conventional research setups. Moreover, even with subsequent task-specific fine-tuning, the generalization capabilities learned during pre-training can degrade, limiting the practical utility of these models in diverse real-world scenarios.

Current VLA training pipelines typically consist of two stages: (1) pre-training a VLA model using massive web data and diverse human demonstrations on a pre-trained vision-language model, followed by (2) supervised fine-tuning with limited task-specific or embodiment-specific data. Recent studies (Zhang et al., 2026; Li et al., 2025b; Lu et al., 2025; Turcato et al., 2025) have explored Reinforcement Learning (RL) during the fine-tuning phase, demonstrating that RL can outperform purely demonstration-based supervision. However, these works are still limited to task-specific fine-tuning and still rely on costly, large-scale pre-training datasets, which face a critical challenge: the pre-training phase requires extensive human and equipment resources to acquire massive demonstration data, which is prohibitively expensive for conventional research. Moreover, the generalization capabilities developed during pre-training inevitably degrade after task-specific fine-tuning. While task-specific fine-tuning remains indispensable for achieving strong performance in practical robotic manipulation, it is imperative to explore more efficient pre-training methodologies that reduce heavy reliance on large-scale demonstration data for VLA models.

**Figure 1:**
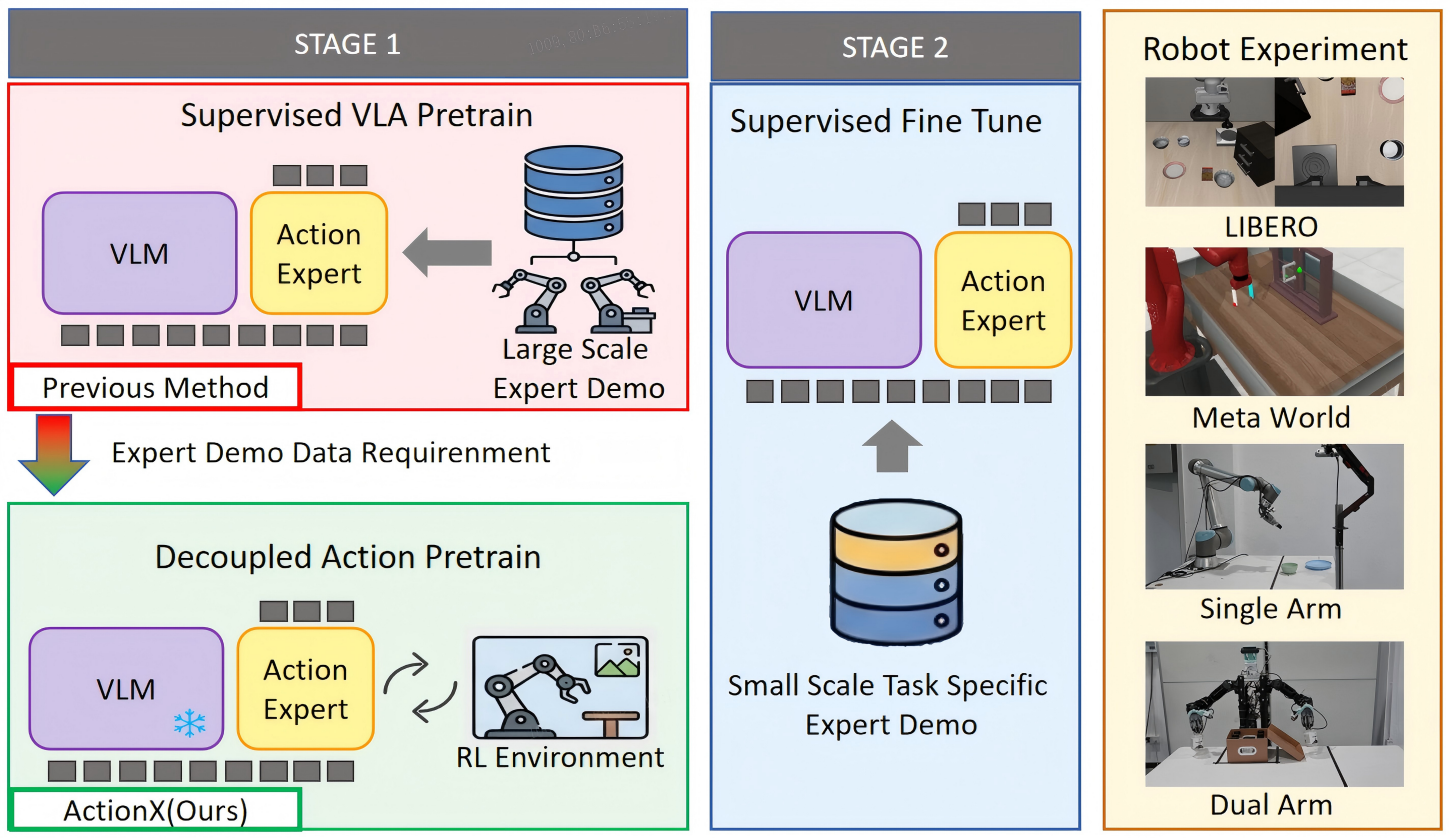
ActionX: an action expert pre-train approach for VLA model. ActionX leverages reinforcement learning to conduct data efficient training on both simulation and real robot on manipulation tasks without using large scale expert demonstration data as other supervised pre-train approach did.

To address this challenge, we propose ActionX, as illustrated in Figure 1, a two-stage RL-based pre-training strategy. Our key insight is that, given the inevitability of task-specific fine-tuning, decoupling the pre-training of the VLM and action expert yields greater benefits than joint optimization. Since pre-trained VLMs already provide strong generalization, we focus on pre-training the action expert with task-specific action distributions to build an effective foundation for rapid downstream fine-tuning.

Our two-stage approach first freezes the VLM backbone and trains the action expert via RL in the first stage, followed by joint full-parameter supervised fine-tuning on task-specific data in the second stage. Unlike existing methods, the first stage focuses exclusively on reinforcing the action expert, enabling performance comparable to fully pre-trained VLA models while substantially reducing reliance on massive web data and human demonstration datasets. We validate our method on the challenging benchmarks LIBERO (Liu et al., 2023) and Meta-World (Yu et al., 2020), as well as real-robot experiments. Results demonstrate that our ActionX significantly improves task success rates in robotic manipulation. Physical robot experiments further confirm that our approach achieves an average 16% higher task success rate in long-horizon operations compared to baseline methods without requiring large-scale data at any stage of training.

Our contributions are summarized as follows:

1. We introduce ActionX, a two-stage VLA training framework that first pre-trains the action expert using reinforcement learning with a frozen VLM, followed by full-parameter fine-tuning to adapt for specific robotic manipulation tasks.
2. We propose an RL-based pre-training strategy that efficiently aligns the action expert with task-specific distributions without requiring extensive manipulation demonstrations.
3. We demonstrate that ActionX achieves state-of-the-art performance both on LIBERO and Meta-World benchmarks and on real robots using only a small batch of expert data.

## 2 Related work

### 2.1 Multimodal robotic skill

Multimodal robotic skill has become a new pivot in embodied intelligence, enabling complex manipulation by integrating visual perception, robotic motion planning and other potential modalities. In recent years, various approaches have emerged in manipulation field, imitation learning methods (Fu et al., 2024; Chi et al., 2025; Zhu et al., 2023; Shah et al., 2025) have achieved dexterous manipulation in table top tasks by enabling robots to imitate demonstrated actions. On the other hand, extensive research has attempted to fuse multiple modalities to guide robot actions (Mehri Shervedani et al., 2025; Zhao et al., 2022). Beyond vision, researchers have explored incorporating auditory (Zhao et al., 2022), force (Yu et al., 2025), tactile (Zhang Z. et al., 2025), and other sensory modalities to guide robot behaviors. More recently, building upon the multimodal perception and analysis capabilities of Vision-Language Models, a strategy incorporating robotic control mechanisms has emerged as an effective approach (O'Neill et al., 2024; Kim et al., 2024; Black et al., 2024; Liu et al., 2024). By expanding Action Expert models, these methods achieve autonomous robot control based on visual and language inputs. However, these works primarily rely on supervised learning with large-scale datasets in terms of training strategies. In contrast, our work focuses on developing efficient action expert training methods that reduce dependency on massive data collection. Beyond improving action expert models, another line of research aims to enhance overall VLA capabilities through internet-scale data and synthetic data (Bjorck et al., 2025; Li et al., 2025a). While these approaches partially alleviate the challenges of data collection, they impose high demands on computational resources. In comparison, our work reduces computational resource dependency by minimizing the data volume required for training. Additionally, further works (Wen et al., 2025; Shukor et al., 2025) have improved VLA inference speed through lightweight VLM model architectures from the perspective of inference acceleration. Building upon this lightweight design philosophy, our work further investigates how to enhance overall robot control performance through action expert optimization, meanwhile reducing training resource requirements.

### 2.2 Reinforcement learning for multimodal robotic tasks

Constrained by the high costs of data collection, how to efficiently train high-performance multimodal robotic skill models has remained an important topic. Reinforcement learning has demonstrated strong potential in this domain, showing promising applications in various robotic tasks (Mehri Shervedani et al., 2025; Li and Zhou, 2023; Zhao et al., 2022). Recent works (Zang et al., 2025; Lu et al., 2025; Li et al., 2025b) have established foundational reinforcement learning platforms for subsequent research by combining diverse simulation benchmarks (Liu et al., 2023; Yu et al., 2020) with multimodal robotic control models pre-trained by supervised learning. Further research (Chen et al., 2025) explored how common action expert mechanisms can collaborate with reinforcement learning, establishing feedback strategies adapted to flow matching. It deeply discussed the advantages of using reinforcement learning for post-training pre-trained multimodal robotic control models, inspiring our consideration of using similar mechanisms for pre-training. Meanwhile, VLA-RL (Lu et al., 2025) proposed strategies for learning under sparse rewards across multiple tasks, but it is limited to simulation environments. SimpleVLA-RL (Li et al., 2025b) validates the model trained by reinforcement learning in real-world environments through sim2real transfer, demonstrating the feasibility of reinforcement learning in real robot experiments. Further work GR-RL (Li Y. et al., 2025) proposed use both offline and online reinforcement learning in different stage, which showed us the potential of multi-stage training framework. Additionally, HIL-SERL (Luo et al., 2025), VLAC (Zhai et al., 2025) and $\pi_{0.6}^{*}$ (Amin et al., 2025) explored real-world robot reinforcement learning with human correction, providing valuable references for our real-robot research. Overall, reinforcement learning can effectively address the challenges of training data collection in multimodal robotic control across various robotic tasks. However, such research has primarily focused on post-training after supervised learning. In contrast, our exploration of leveraging reinforcement learning for pre-training offers a novel perspective for related research.

## 3 Methodology

### 3.1 Problem formulation

We model our approach using a Markov Decision Process (MDP) defined by a tuple $\left(S,A,P_{0},P_{\text{ENV}},R,\gamma \right)$, in which the state $s_{t}\in S$ at time *t* is composed of vision language observation data $o_{t}^{VL}$ and robot proprioceptive state $o_{t}^{R}$. *P*_0_ represents the initial state distribution. Given a state *s*_*t*_, an action $a_{t}\in A$ is sampled from a policy π(·∣*s*_*t*_) that induces a state transition *s*_*t*+1_~*P*_ENV_(·∣*s*_*t*_, *a*_*t*_) and yields an immediate reward *R*(*s*_*t*_, *a*_*t*_). For reinforced pre-training stage, the goal is first to learn an optimal action distribution that maximizes the expected cumulative reward under the discount factor γ over a horizon of *T*. For the second stage of finetuning, the policy π_θ_ is optimized for specific task through supervised learning by leveraging expert demonstrations $D=\left\{\left\{o_{1}^{VL},o_{1}^{R},a_{1}\right\},...,\left\{o_{T}^{VL},o_{T}^{R},a_{T}\right\}\right\}$.

### 3.2 ActionX framework

**Figure 2:**
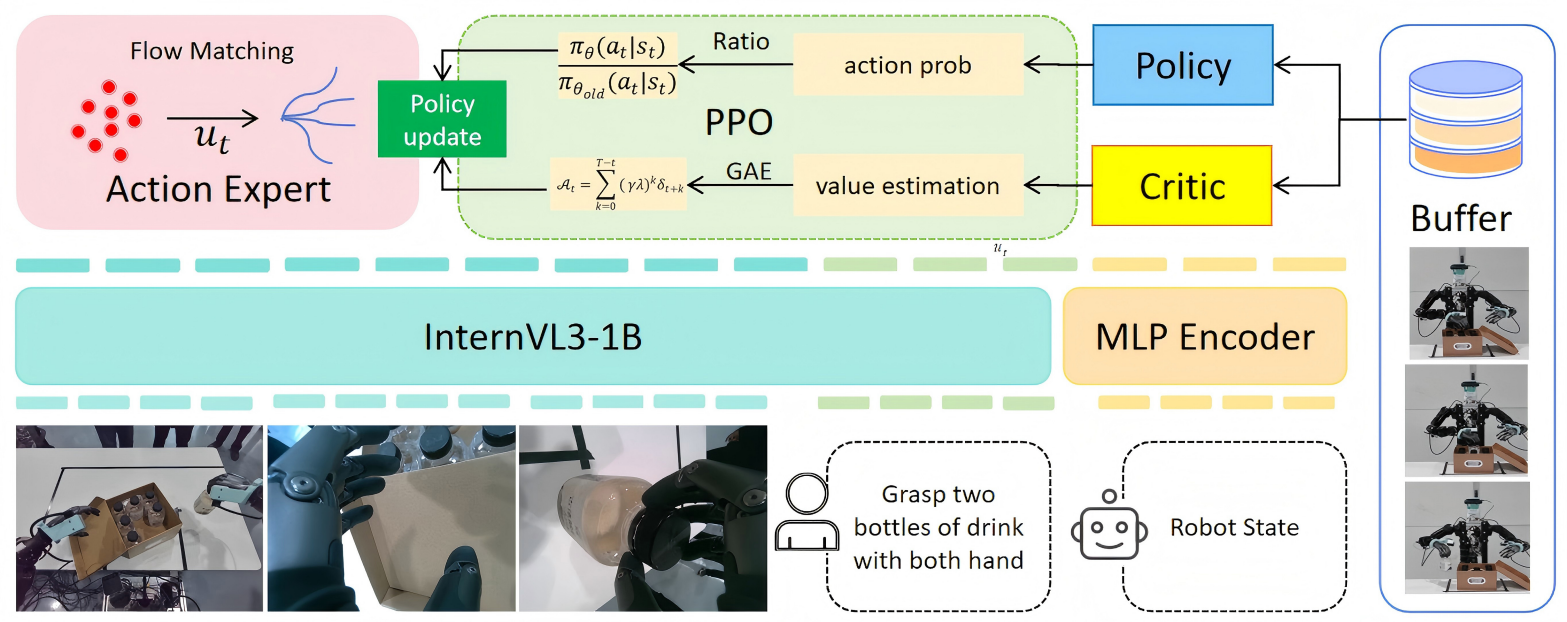
Overview of ActionX: our approach combine fused visual-language input and robot state embedding to support a flow based action expert perform manipulation reinforcement learning with actor-critic framework. The actor and critic collect reward and update their self at the end of each action chunk generated by action expert.

We propose ActionX, a decoupled action expert pre-training framework with reinforcement learning, which consists of two stages: (1) pre-training an action expert with reinforcement learning to model the action-space distribution; and (2) jointly fine-tuning the VLM and the action expert on a specific downstream task. As shown in Figure 2, ActionX adopts a model architecture that combines a flow matching based action expert with a pre-trained VLM backbone. The action expert predicts continuous actions *a*_*t*_ for a robot in specific states through a denoising process, while a VLM backbone collaborates with a robot state encoder to estimate the current latent condition that guides the action expert, based on multimodal observations.

We implement the action expert as an 8-layer self-attention transformer model with a sinusoidal time step encoder to learn the connection between a noise distribution and the true action distribution of robot tasks, via linear interpolation over time. The action expert is conditioned on a latent representation *z*_*t*_, which is formed by concatenating the hidden state of the VLM with an embedding of the robot state. The goal of the action expert model is to predict a time-dependent velocity field $v_{\theta }\left(A_{t}^{\tau },s_{t}\right)$ that approximates the target velocity field, where $A_{t}^{\tau }$ is a noisy action sequence obtained by weighting a randomly sampled noise vector ϵ~*N*(0, 1) and the ground truth action sequence *A*_*t*_ using weight τ drawn from a Beta distribution:


$$A_{t}^{\tau }=\tau A_{t}+\left(1-\tau \right)\epsilon .$$

During inference, the algorithm starts from $A_{t}^{0}$ and iteratively generates an action sequence according to the following formula:


$$A_{t}^{\tau +dt}=A_{t}^{\tau }+dt\cdot v_{\theta }\left(A_{t}^{\tau },s_{t}\right).$$

For observation state, we use a pre-trained vision-language backbone model InternVL3-1B (Zhu et al., 2025), combined with its distilled visual encoder InterViT-300M, as the preprocess tool for visual and linguistic modalities in observations. The relatively small number of parameters and the efficient feature fusion capability of InternVL3-1B allow effective training on diverse tasks. By inserting features of image patches into the token sequence and uniformly decoding them using a Transformer, this strategy achieves fusion of visual and linguistic context within a unified embedding space. Inspired by SmolVLA (Shukor et al., 2025), we only use the first half of the language model to extract a more robust global state representation based on the visual and linguistic inputs directly observed at time *t*. For the robot state $o_{t}^{R}$, we use an MLP encoder to compute its embedding, which is then concatenated with the hidden state of the vision-language model:


$$z_{t}=\phi_{vlm}\left(o_{t}^{VL}\right)⊕\phi_{mlp}\left(o_{t}^{R}\right)$$

Different from other methods such as π_0.5_ (Intelligence et al., 2025) that directly fuse robot state embeddings into multi-modal features, our approach uses the original robot state information for action generation. Since actions are strongly correlated with robot states, maintaining an independent robot state embedding helps the model capture this correlation more effectively during training and accelerates the convergence speed.

### 3.3 Action pre-train with reinforcement learning

A major challenge in training VLA models is how to efficiently compute the robot's action distribution while aligning VLM model with the conditional context of flow matching. Typically, supervised learning approaches (Bjorck et al., 2025; Intelligence et al., 2025) adopt a strategy of jointly fine-tuning the VLM and the action expert to achieve optimal performance. However, this method requires a large amount of data and high computational costs. To address this, we propose a pre-training strategy that accelerates the overall training process and improves final success rate by learning a better-initialized action distribution. Our approach leverages reinforcement learning to pre-train the action expert with a frozen VLM backbone, followed by joint full-parameter fine-tuning of both components.

A key challenge when applying reinforcement learning process with the flow matching mechanism is that the action expert outputs an action chunk corresponding to a sequence of continuous actions during each inference step. This action chunk corresponds to multiple discrete steps in the RL process, thus leading to multiple environment feedbacks per inference step. To address this, we design a learning strategy based on PPO (Schulman et al., 2017) that updates only at the action chunk level. For the Actor model, complete inference is performed only during the computation of each action chunk. Here, we use generalized advantage estimation based on TD-error δ_*t*_ to calculate low-variance advantage *A*_*t*_:


$$\delta_{t}=R_{t}+\gamma V\left(z_{t+1}\right)-V\left(z_{t}\right),$$

$$A_{t}=\overset{T-t}{\underset{k=0}{\sum }}{\left(\gamma \lambda \right)}^{k}\delta_{t+k},$$

where $R_{t}=\overset{H-1}{\underset{j=0}{\sum }}r_{t,j}$ is the cumulative reward of an action chunk, γ is the discount factor, λ is used to balance variance and bias during advantage estimation, and *V*(·) denotes the state value function computed by the critic. Finally, we update the actor model using the following loss function:


$$\rho =\frac{\pi_{\theta }\left(a_{t}|s_{t}\right)}{\pi_{old}\left(a_{t}|s_{t}\right)},$$

$$L^{\rho }=E_{t}\left[min\left(\rho A_{t},clip\left(\rho ,1-\epsilon ,1+\epsilon \right)A_{t}\right)\right].$$

For the critic model, similar to other VLA implementations (Zang et al., 2025; Li et al., 2025b) using PPO, we employ a structure identical to the actor to ensure relative stability of the training process. The critic is updated after each action chunk. The reward is zero at all intermediate timesteps within a chunk and non-zero only at the final step of the chunk, where the accumulated return is computed.

Additionally, to prevent critic network from misleading actor model during early training stages, we adopt critic warm up method introduced in VLA-RL (Lu et al., 2025). Specifically, we initialize the critic network by training it for several iterations using rollouts of a frozen actor policy that was pre-trained via imitation learning on limited demonstration data, to learn accurate state value estimates before proceeding standard joint actor-critic training. In practice, the warm up phase typically utilizes 10% of total demonstration data that used in subsequent SFT phase, thus requiring no additional demonstrations. This approach establishes critic network's ability to evaluate state values, thereby reducing the learning complexity of sparse reward long-horizon tasks.

### 3.4 Supervised fine tune

After completing the reinforcement learning pre-training of the model, the action expert model has preliminarily acquired the action distribution state required for the robot. At this stage, further full-parameter fine-tuning will converge faster than directly training the VLM backbone and action expert jointly. In some long-horizon tasks, this approach improves the overall learning capability and efficiency of the model.

We use expert demonstration data for supervised fine-tuning, where the velocity field of action distribution change $v_{\theta }\left(A_{t}^{\tau },s_{t}\right)$ is trained to approximate the ground-truth results derived from robot state sequences in expert data, denoted as $u\left(A_{t}^{\tau }|A_{t}\right)=\epsilon -A_{t}$, as shown in formula below:


$$L^{\theta }=E_{p\left(A_{t}|s_{t}\right),q\left(A_{t}^{\tau }|A_{t}\right)}||v_{\theta }\left(A_{t}^{\tau },s_{t}\right)-u\left(A_{t}^{\tau }|A_{t}\right)|{|}^{2}$$

Using the prior pre-training of the action expert model and its initial alignment with the task-specific action distribution, the training process can more quickly enter the stage of fine-grained action tuning. Additionally, because the action distribution shares attention mechanisms with the default VLM, the initial alignment between the two components accelerates the convergence of the VLM component during subsequent training.

## 4 Evaluation

### 4.1 Simulation experiments

In this study, we use the success rate of tasks as the primary evaluation metric. We adopt two widely used robotic manipulation benchmark datasets: LIBERO (Liu et al., 2023) and Meta-World (Yu et al., 2020). The LIBERO dataset contains multiple subsets, where subset Goal focuses on goal centered semantic tasks, subset Object emphasizes object-centric manipulation tasks, subset Spatial involves spatial relation reasoning tasks, and subset Long targets long-horizon, multi-stage tasks. These subsets collectively enable comprehensive evaluation of models' generalization capabilities in diverse and semantically rich daily manipulation scenarios. To systematically measure model performance, we follow previous research (Shukor et al., 2025) select 10 representative tasks from each sub-dataset of LIBERO, covering different semantic instructions, object configurations, and environmental layouts. For each task, we independently run 10 evaluation trials and record whether the task is successfully completed, ultimately using the task success rate as the core evaluation metric. The observation space, action space and reward used in this experiment are set as follows:

- ***Observation**:* 640 × 480 images from wrist and third person view cameras with normalized absolute robot arm joint state and gripper state.
- ***Action**:* normalized absolute robot arm joint state and gripper state.
- ***Reward**:* sparse 0/1 reward used across all tasks, where reward is 1 only upon task success and 0 otherwise.

Additionally, we introduce the Meta-World benchmark, which provides 50 simulated robotic control tasks with distinct reward structures, further validating the models' ability. In Meta-World, we similarly perform 10 independent tests for each task, again using success rate as the primary comparison basis. The average success rate is computed as the mean of the success rates across the four difficulty categories, consistent with the methodology used in other studies (Shukor et al., 2025). The observation space, action space and reward used in this experiment are set as follows:

- ***Observation**:* 640 × 480 images from third person view cameras with normalized absolute robot gripper Cartesian pose and gripper state.
- ***Action**:* normalized absolute Cartesian pose and gripper torque.
- ***Reward**:* sparse 0/1 reward used across all tasks, where reward is 1 only upon task success and 0 otherwise.

Regarding baselines, we use Diffusion Policy (Chi et al., 2025), OpenVLA (Kim et al., 2024), π_0_ (Black et al., 2024), π_0_-FAST (Pertsch et al., 2025), SmolVLA (Shukor et al., 2025), and TinyVLA (Wen et al., 2025) as baseline methods. Diffusion Policy is the first framework adopting diffusion models for action generation in policy imitation learning, while OpenVLA represents an early implementation of VLA models. π_0_ and its accelerated version π_0_-FAST are widely used VLA baseline frameworks that significantly enhance VLA capabilities through flow-matching based action generation and cross attention mechanisms. TinyVLA and SmolVLA achieve high-speed inference through efficient VLM backbones and layer-skipping techniques, respectively. Currently SmolVLA represents the recent state-of-the-art method, achieving high-performance action reasoning with a compact VLM backbone.

On both datasets, we compare against Diffusion Policy, π_0_, π_0_-FAST, and SmolVLA to analyze performance differences in both single-step and long-horizon tasks. Besides, we add OpenVLA tests on LIBERO and TinyVLA on Meta-World to better evaluate the result.

**Figure 3:**
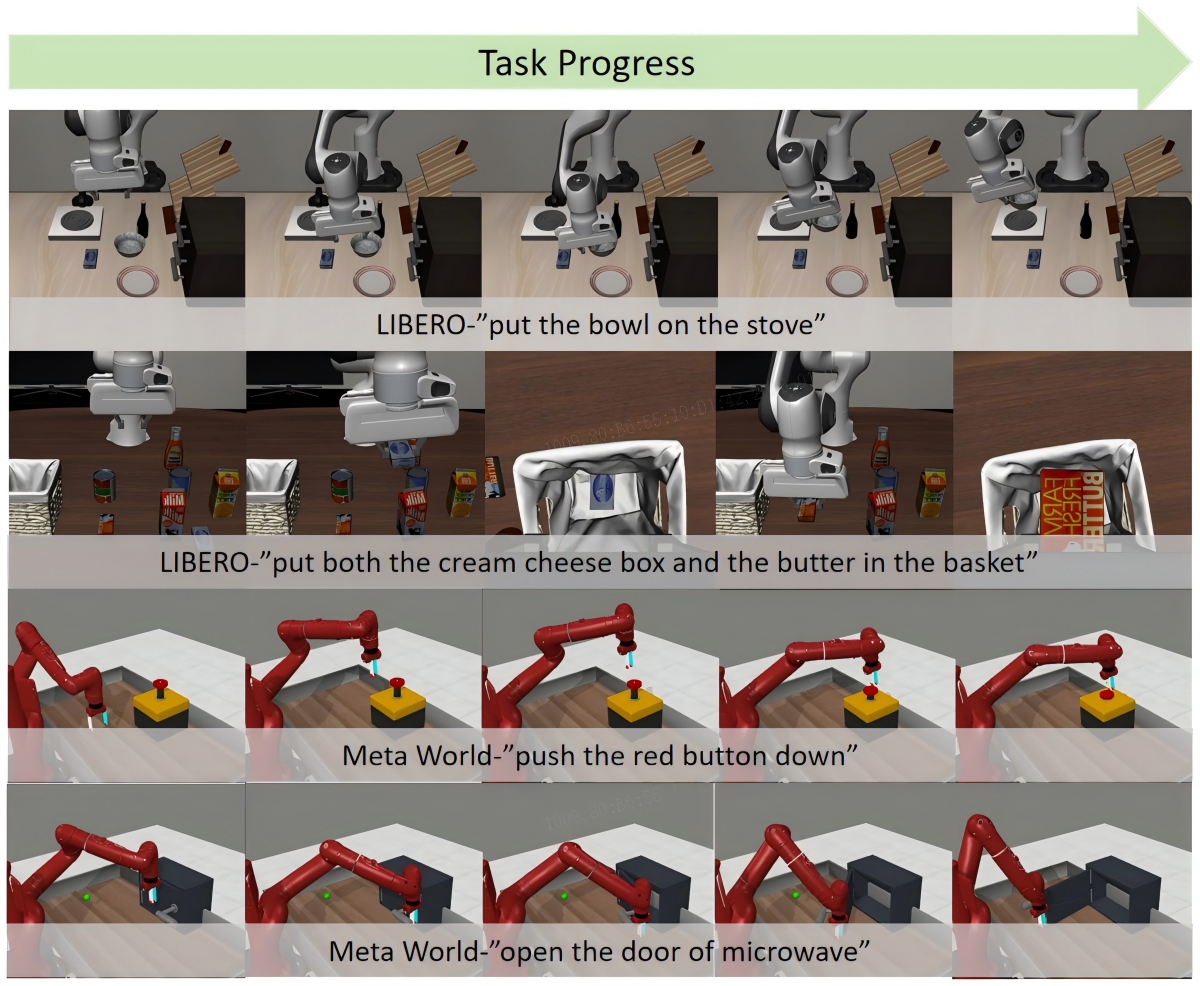
Long horizon robot manipulation in LIBERO and meta-world simulation.

Our evaluation uses all 4 LIBERO subsets and 50 tasks in Meta-World as illustrated in Figure 3 with 10 repetitions per task. We compare different Vision-Language-Action models across multiple tasks including pick and place, goal reaching, and long horizon task handling.

For LIBERO experiments, we pre-train the action expert for 20,000 RL steps, and warm up our critic network for all 4 subsets respectively using 10% data of its human teleoperation dataset. Besides, we also fine tuned all the models with LIBERO's whole dataset on each subset, to better compare the maximum performance of each model. For Meta-World experiments, we generate 50 demonstrations of each task by its built-in expert policies, and apply similar training configuration as LIBERO experiments used for our approach and all the baselines. These two datasets together form a robust evaluation platform for assessing the comprehensive capabilities of various VLA models.

As shown in Table 1, in the experiments on the LIBERO dataset, our method achieves an average success rate of 91.5%, surpassing the previous state-of-the-art method SmolVLA.

**Table 1:** Performance comparison on LIBERO and Meta-World benchmarks.

Benchmark	Policy	Success rate (%)				Avg.
		Goal	Object	Spatial	Long	
LIBERO	Diffusion policy	78	92	68	50	72.0
	OpenVLA	79	88	85	54	76.5
	π_0_	90	86	95	73	86.0
	SmolVLA	91	94	93	77	88.8
	**Ours**	**94**	**94**	**95**	**83**	**91.5**
		Easy	Medium	Hard	Very hard	
Meta-world	Diffusion policy	23.1	10.7	1.9	6.1	10.5
	TinyVLA	77.6	21.5	11.4	15.8	31.6
	π_0_	71.8	48.2	41.7	30.0	47.9
	SmolVLA	87.1	51.8	70.0	64.0	68.2
	**Ours**	**90.1**	**74.2**	**79.4**	**81.5**	**81.3**

Bold values represent the success rates of the proposed ActionX method.

Given that prior methods had already achieved high performance on Goal, Object and Spatial subsets, the gains from our method remain relatively modest in these cases. However, on the long-horizon task subset LIBERO Long, our method achieves a success rate of 83%, marking a +6% improvement over SmolVLA. This result is generated from our training strategy, which initially freezes the vision-language model and employs pre-training with reinforcement learning to establish a superior action distribution model early in training. Compared to the three subsets with shorter action sequences, the Long subset exhibits more diverse and complex action distributions. Thus, the pre-training strategy better initializes the action expert module, enabling the VLM to receive more effective feedback from contextually relevant actions during subsequent joint training of the VLM and action expert modules. This mechanism ultimately drives performance breakthroughs in long-horizon tasks.

In the simulation experiments on the Meta-World benchmark, our method achieves approximately +13% improvement in average success rates compared to prior work. The most significant gains are observed on medium and hard sub-datasets, which primarily consists of long-horizon, fine-grained manipulation tasks requiring precise operations. Unlike tasks emphasizing large-scale spatial movements, these tasks demand richer orientation adjustment capabilities, necessitating more complex action distributions.

It is worth noting that experiments on LIBERO and Meta-World have demonstrated that our proposed method, which combines a pre-trained action expert model with a VLM model not specifically trained for robot manipulation, achieves comparable robot manipulation capabilities to VLA models pre-trained with massive expert data. Although other VLA models may exhibit superior generalization abilities, it is critical to consider that all VLA frameworks require full-parameter fine-tuning during practical deployment. This fine-tuning process significantly degrades their generalization performance. Consequently, our method achieves higher success rates in final manipulation tasks compared to existing approaches.

### 4.2 Real robot experiment

To validate the practical applicability of our method in real-world scenarios, we conducted physical experiments on both single-arm and dual-arm robotic configurations. Notably, all experiments required fewer than 80 epochs of expert data for SFT.

For real-robot deployment, we adopted a Client/Server architecture. A VLA inference service hosted on a workstation with RTX A6000 GPU acts as the server, while a lightweight module embedded in the robot controller executes the client. Operating at 20 Hz, the client streams cameras and robot state data to the server, receives computed motion trajectories, and directly actuates the robot accordingly.

**Single-arm robot experiments** utilized a UR12E robotic arm equipped with a two-fingered gripper. The system was supported by two RealSense D435 cameras: one dedicated to third person view data acquisition and the other to close-up visual data from the wrist-mounted perspective. In this experiment, the robot was required to place specified objects onto a round disk. The initial robot position, target grasp location, and disk position were randomized during testing to evaluate robustness. The observation space, action space and reward used in this experiment are set as follows:

- ***Observation**:* 640 × 480 images from both wrist and third person view cameras with normalized absolute robot arm joint state and gripper state.
- ***Action**:* normalized absolute joint position and gripper position.
- ***Reward**:* sparse 0/1 reward used for RL, where reward is 1 only upon task success and 0 otherwise.

**Figure 4:**
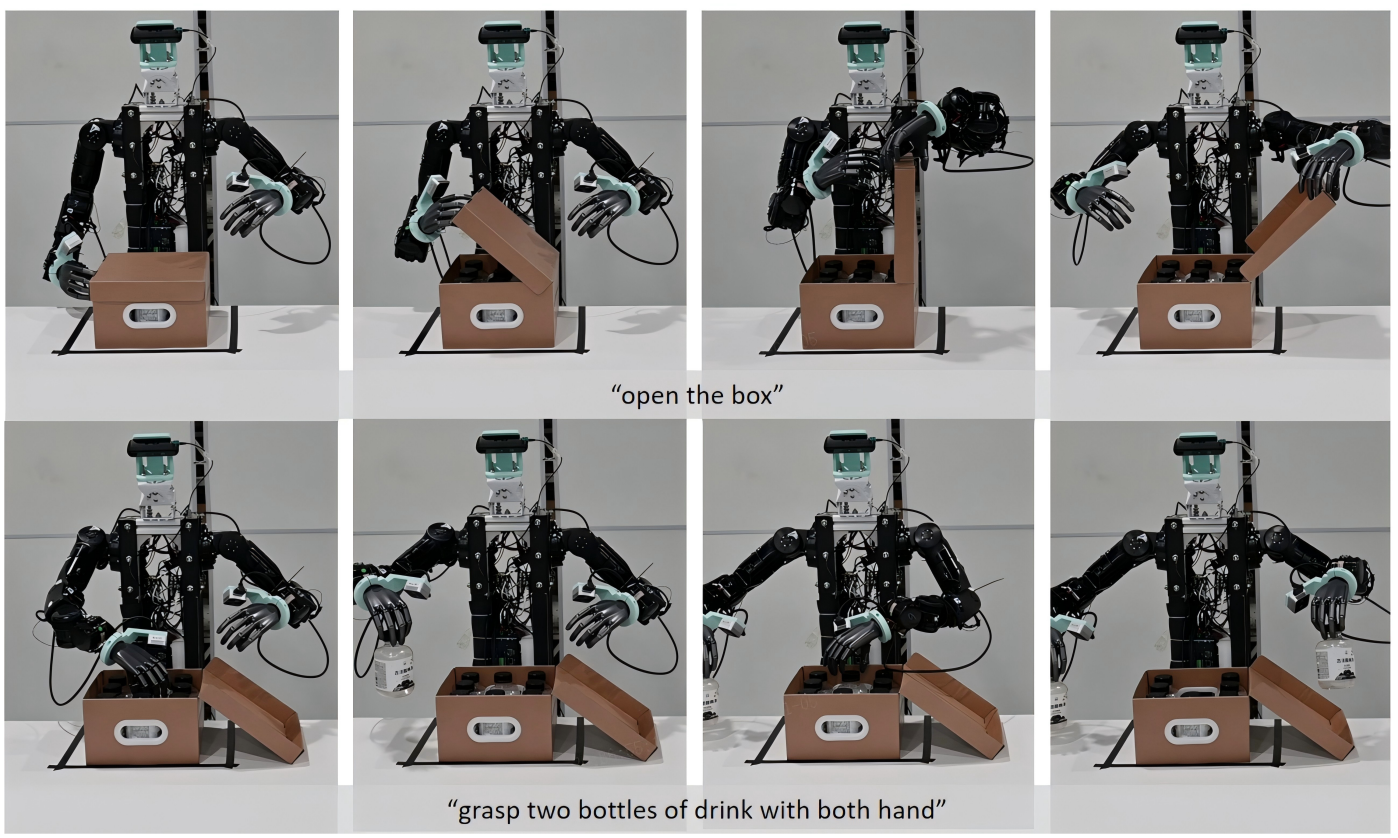
Long horizon tasks on real robot. The robot was asked to open a box first then pick up two bottle of water with both hands.

**Dual-arm robot experiments** employed our custom-built dual-arm platform, where each arm featured 7 degrees of freedom (DoF) and was paired with a 6-DoF robot hand to execute complex tasks. The vision system integrated three cameras: an Orbec Gemini336L mounted on the head and two RealSense D405 cameras positioned at the wrists. The robot was required to complete a long-horizon task involving opening a package box lid and retrieving two bottles of water, as demonstrated in Figure 4. The open box task demanded bimanual cooperation for lid manipulation and the other task required precise manipulation capabilities for grasping. The observation space, action space and reward used in this experiment are set as follows:

- ***Observation**:* 640 × 480 images from all three cameras with normalized absolute joint state of both arms and two integer represent left and right hand state respectively.
- ***Action**:* normalized absolute joint position for both arms and and two integer signals which control robot's both hands switching between 5 predefined gestures.
- ***Reward**:* sparse 0/1 reward used for RL, where reward is 1 only upon task success and 0 otherwise.

We separately measured and reported the success rates of each task. In this experiment, we compared the performance of OpenVLA (Kim et al., 2024), SmolVLA (Shukor et al., 2025), and π_0.5_ (Intelligence et al., 2025) model and our model with identical data. We use PPO with same data to prepare action expert pre-train for our model.

**Figure 5:**
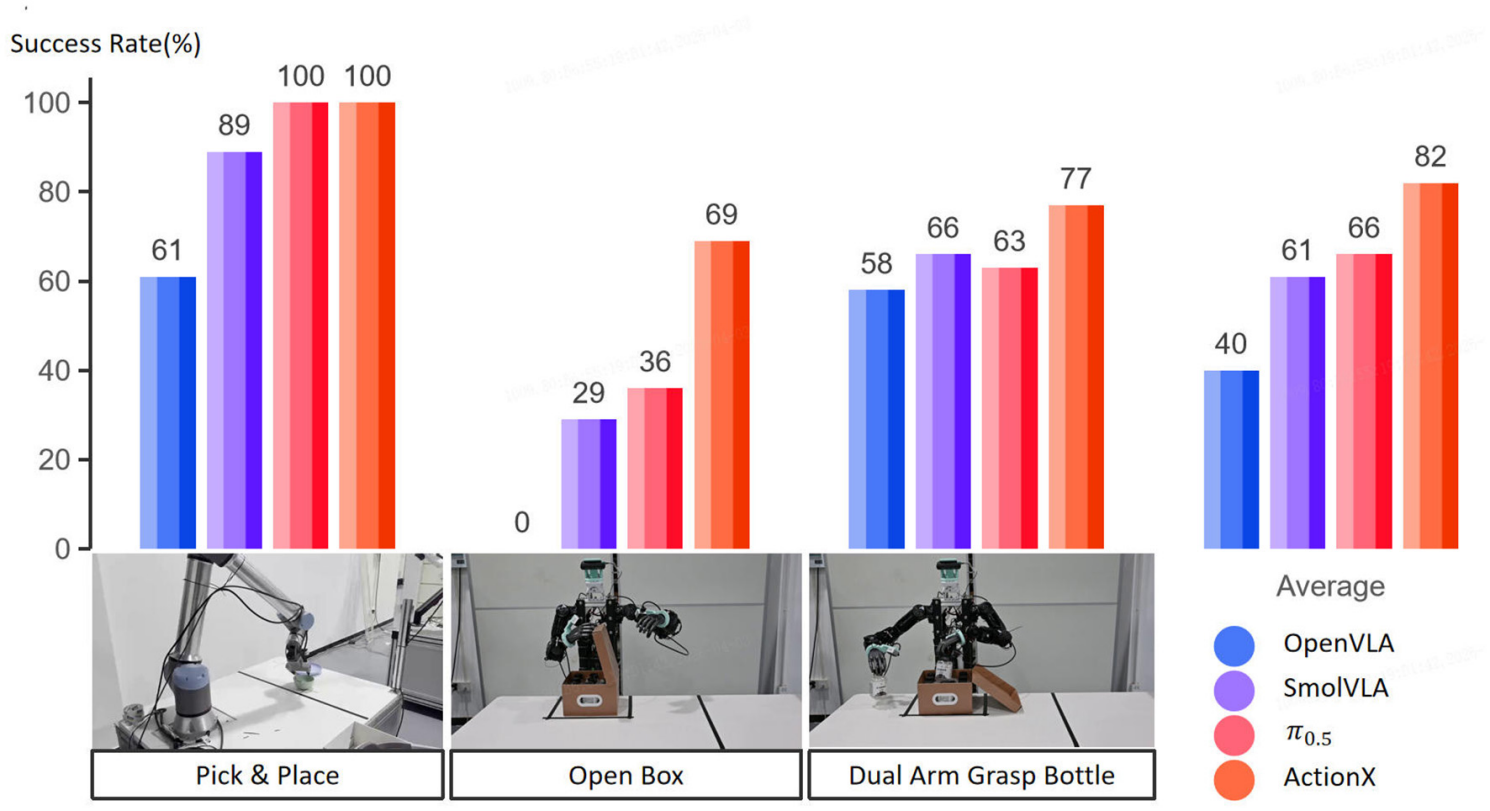
Comparison on real robot manipulation task, our approach present highest success rate, especially in long-horizon task open box, which require cooperation for both arms.

**Figure 6:**
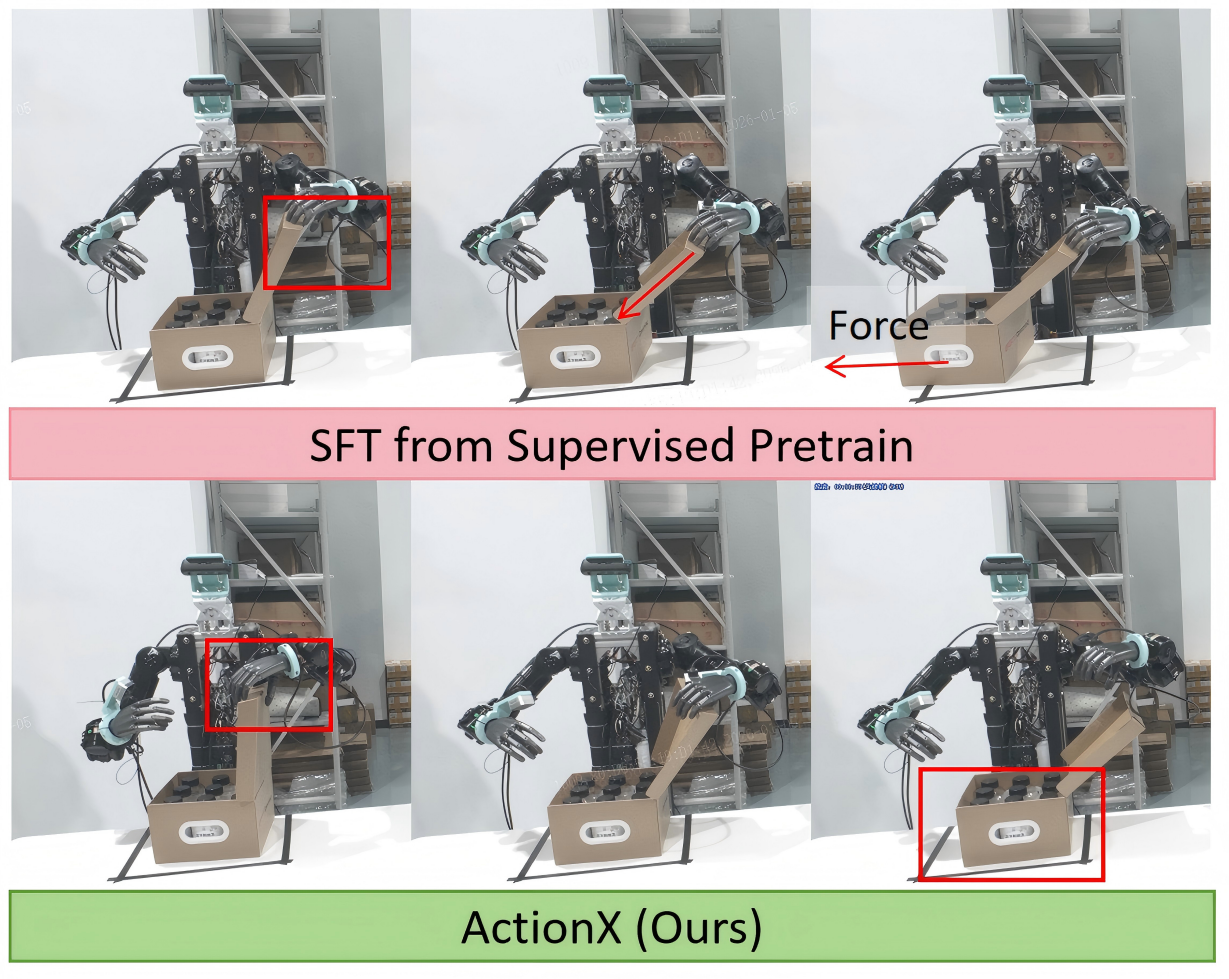
The hybrid force-position actions in teleoperation demonstrations may lead to failures in specific tasks. Our reinforcement learning based pre-training approach can avoid force contact through slight adjustments to wrist orientation, thereby improving task success rates.

It can be observed from Figure 5 that ActionX demonstrates higher success rates in dual-arm tasks. During our analysis, we found that compared to purely supervised learning strategies, our ActionX method enables the model to utilize more diverse action patterns. As illustrated in Figure 6, the expert teleoperation data may contain unintentional hybrid force-position control actions that are challenging for the robot to learn. When executing similar actions using the supervised learning strategy, the robot may push the box away from its original position, leading to unexpected behavior. In contrast, the model pre-trained with reinforcement learning exhibits richer action options, which mitigates the negative impact of complex actions present in the expert demonstration data that are difficult to replicate.

### 4.3 Ablation study

To validate the key design of ActionX, specifically the effectiveness of the reinforcement learning based pre-training strategy for the action expert model, we conduct two ablation experiments. First, we analyze the differences in learning trend between models trained with and without the action expert pre-training strategy. Second, we compare wrist orientation flexibility of robot to discuss generalization ability of different training policies. In both comparisons, we use the original vision-language pre-trained model as the VLM backbone, without VLA-specific pre-training or fine-tuning prior to the experiments. All pre-training methods were trained for 5,000 steps, and the step counts reported in our experiments represent the sum of pre-training and full-parameter fine-tuning steps. Following the evaluation protocol described in Section 4.1, we assess the success rates of the models on simulated tasks at different steps of full-parameter fine-tuning after pre-training. This allows us to analyze how the pre-training phase contributes to the overall training efficiency and performance of the model.

#### 4.3.1 Study on action expert model pre-training

To evaluate the value of the pre-training phase design, we compare RL-based action expert pre-training against supervised pre-training and no action expert pre-training. All the pre-training phases is followed by an identical supervised fine-tuning on four sub-task suites from LIBERO, specifically examining the impact of pre-training phase on the subsequent convergence speed of the later full parameter tuning stage. In our experiments, the critic warm-up phase of RL only uses 10% of whole expert demonstration data and the supervised learning phase utilizes the complete dataset. As shown in Table 2, results demonstrate that employing an RL-based pre-training strategy significantly accelerates early-stage convergence and also yields a modest improvement in final success rates under identical training conditions.

**Table 2:** Ablation on action expert pre-train.

Benchmark	Pre-train method			Success rate over steps				
	None	SFT	RL	5k	10k	20k	30k	40k
LIBERO-goal	✓			17	33	71	84	85
		✓		25	47	67	85	96
			✓	39	72	91	94	94
LIBERO-object	✓			7	31	80	89	91
		✓		23	46	80	87	94
			✓	29	58	83	89	94
LIBERO-spatial	✓			0	0	0	0	0
		✓		43	47	80	89	92
			✓	45	61	79	93	95
LIBERO-long	✓			1	1	3	6	14
		✓		1	5	23	41	52
			✓	5	19	31	47	66

Notably, in comparison with direct supervised learning, experiments on the spatially oriented subset Spatial reveal that directly fine-tuning from a pre-trained VLM backbone with supervised learning fails to learn an effective action policy. In contrast, our pre-training approach dramatically resolves this issue, boosting success rates from 0% to 95%. Furthermore, on the subset Long, which involves long-horizon tasks which commonly suffer from compound error problem, our method achieves a performance gain of over +50% in task success rate. These findings indicate that standard VLM backbones possess limited capability in spatial action reasoning and long-horizon action planning, without richer feedback from an action expert to effectively adapt to Vision-Language-Action tasks. By leveraging pre-training with reinforcement learning, the action expert learns a more robust prior over fundamental action distributions. Consequently, during the subsequent joint training phase between the VLM backbone and the action expert, this improved action prior enables more accurate feedback for VLM, thereby accelerating overall convergence and enabling successful learning of complex tasks that are infeasible under purely supervised fine-tuning.

#### 4.3.2 Study on training cost over task diversity

The training of VLA models scales with both task complexity and the volume of data utilized. To further analyze the training scalability of the ActX framework, we conducted experiments using robot simulation data from the LIBERO benchmark, investigating how the number of steps required for convergence changes as the data scale increases. Furthermore, we compared the convergence steps between training on single task type vs. training on multiple mixed tasks.

Our experiments utilized three specific subsets from the LIBERO dataset: Goal, Object, and Spatial, along with a Mix dataset formed by combining these three groups. Initially, we performed reinforcement learning pre-training for each model using its corresponding simulation environment; notably, the model for the Mix dataset was pre-trained simultaneously across all three simulation environments. Subsequently, we evaluated supervised fine tuning process using four sub-datasets containing varying percentages of the original data: Tiny (10%), Small (25%), Medium (50%), and Complete (100%). We report the training steps required for convergence during both the simulation RL pre-training stage and data SFT stage. We established a convergence threshold of 10^−3^ for the pre-training stage and 10^−4^ for the fine-tuning stage. Convergence is defined as the iteration step at which the change in loss remains below the specified threshold for 20 consecutive iterations.

The experimental results, presented in Table 3, reveal a strong correlation between the number of training steps required by our method and task complexity. Specifically, both the pre-training and fine tuning phases for the Mix dataset required significantly more steps to converge compared to subsets involving single task types. This increase is attributed to the increased complexity of action space introduced by mixing diverse task types. Consequently, the action expert requires more steps during pre-training to master this expanded space. Furthermore, during supervised fine tuning, the model must additionally learn to process a wider variety of observations, leading to a substantial rise in the total number of training steps. In contrast, the increase in training steps resulting from variations in data volume was not significant. This phenomenon likely stems from the high similarity among tasks within the same dataset. Thus, scaling up the data volume did not substantially enhance data diversity. As a result, the policies required to be learned remained unchanged despite the increased data size, allowing the overall convergence speed to remain relatively stable.

**Table 3:** Ablation on convergence steps of different training settings.

Task	RL-pre-train	Fine tune data			
		Tiny	Small	Medium	Complete
Goal	5,691	26,043	26,512	28,923	30,012
Object	6,128	27,079	29,107	31,677	32,431
Spatial	7,317	27,845	29,563	31,509	32,417
Mix	13,924	42,315	43,290	45,678	49,821

#### 4.3.3 Study on wrist orientation flexibility

During real robot training, teleoperation data collection process may lead the robot to favor fixed postures for task execution, as human operators typically minimize unnecessary pose adjustments to improve efficiency, resulting in lack of samples with diverse pose variations in the dataset for specific task. Nevertheless, our RL-based approach enables the robot to explore a broader range of wrist pitch and yaw angles.

**Figure 7:**
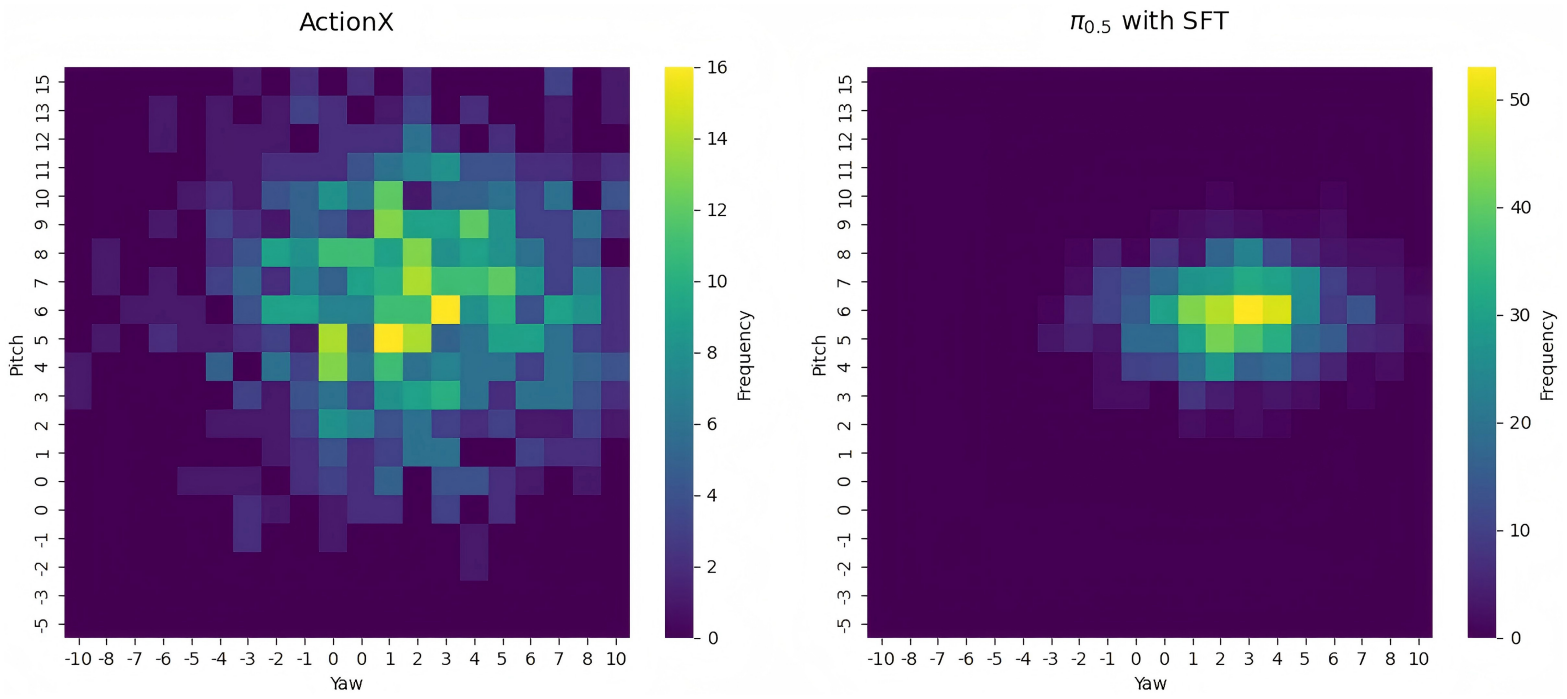
The wrist orientation variance of key action chunks. Our RL based approach present a more flexible wrist orientation, which may benefit dexterous manipulation.

We analyzed the distribution of wrist pitch and yaw angles, in the wrist coordinate frame for key action chunks where the robot's hand makes contact with the target object during the “open box” task, comparing models trained solely via SFT on teleoperation data versus our proposed method. By computing the frequency distribution of different pitch–yaw angle combinations in wrist orientation of each action step from key chunks in valid experiments, we could compare the wrist flexibility of both training strategies. As shown in Figure 7, our method yields a wider distribution of wrist poses during these critical action chunks, indicating that the robot actively explores diverse postures to accomplish the manipulation task. This enhanced flexibility at the pose level, when amplified through hand kinematics, potentially enables the robot to adopt more effective strategies to fulfill the commanded task.

## 5 Conclusion

In this study, we propose ActionX, a new training strategy for Vision-Language-Action models that achieves state-of-the-art performance without requiring large-scale expert demonstration data. This advancement benefits from our action expert pre-training paradigm, which decouples the VLM backbone and action expert module during pre-training to learn task level generalized action distributions. Simulation and real-robot experiments demonstrate that our method significantly improves task success rates in long-horizon manipulation tasks, validating its effectiveness. Future work should focus on enhancing the generalization capabilities of action learning during pre-training, similar to how infants learn to control their limbs before performing meaningful tasks, to develop general purpose action expert models for single or multiple embodied agents.

## Data Availability

The raw data supporting the conclusions of this article will be made available by the authors, without undue reservation.
